# Methodology for sampling women at high maternal risk in administrative data

**DOI:** 10.1186/s12884-019-2500-7

**Published:** 2019-10-21

**Authors:** Jennifer Vanderlaan, Anne Dunlop, Roger Rochat, Bryan Williams, Susan E. Shapiro

**Affiliations:** 1 Emory University Nell Hodgson Woodruff School of Nursing, 1520 Clifton Road, Atlanta, GA 30322 USA; 2 Emory University Rollins School of Public Health, 1518 Clifton Road, Atlanta, GA 30322 USA

**Keywords:** Maternal morbidity, Pregnancy, high-risk, Comorbidity, International classification of diseases, Risk assessment

## Abstract

**Background:**

In population level studies, the conventional practice of categorizing women into low and high maternal risk samples relies upon ascertaining the presence of various comorbid conditions in administrative data. Two problems with the conventional method include variability in the recommended comorbidities to consider and inability to distinguish between maternal and fetal risks. High maternal risk sample selection may be improved by using the Obstetric Comorbidity Index (OCI), a system of risk scoring based on weighting comorbidities associated with maternal end organ damage. The purpose of this study was to compare the net benefit of using OCI risk scoring vs the conventional risk identification method to identify a sample of women at high maternal risk in administrative data.

**Methods:**

This was a net benefit analysis using linked delivery hospitalization discharge and vital records data for women experiencing singleton births in Georgia from 2008 to 2012. We compared the value identifying a sample of women at high maternal risk using the OCI score to the conventional method of dichotomous identification of any comorbidities. Value was measured by the ability to select a sample of women designated as high maternal risk who experienced severe maternal morbidity or mortality.

**Results:**

The high maternal risk sample created with the OCI had a small but positive net benefit (+ 0.6), while the conventionally derived sample had a negative net benefit indicating the sample selection performed worse than identifying no woman as high maternal risk.

**Conclusions:**

The OCI can be used to select women at high maternal risk in administrative data. The OCI provides a consistent method of identification for women at risk of maternal morbidity and mortality and avoids confounding all obstetric risk factors with specific maternal risk factors. Using the OCI may help reduce misclassification as high maternal risk and improve the consistency in identifying women at high maternal risk in administrative data.

## Background

As part of a project examining the effects of regionalization of maternal care, we realized there was no standard for identifying a sample of women likely to be transferred to higher acuity care based on risks to maternal health. Though various research definitions of obstetric risk exist, these often reflect fetal risks or are based on maternal social and medical conditions that do not necessarily indicate a need for higher acuity care to protect maternal health [1–3]. Clinical guidelines, though useful in practice, may not be appropriate for research sample selection because they 1) do not differentiate between maternal and fetal risk, 2) focus on identifying women at low risk rather than defining high maternal risk, and 3) rely on clinical information not available in administrative data [4, 5]. We were concerned that any conventional sample selection method we used would overestimate the clinical maternal risk, causing misclassification bias in our study.

In 2013, Bateman and colleagues proposed a comorbidity summary measure weighted specifically for association between maternal comorbidities and end organ damage or mortality during delivery hospitalization [6]. A list of the included comorbidities is available in Table2. The obstetric comorbidity index (OCI) improves the precision of risk identification by assigning weight to each condition to account for the complexity of multiple conditions. Comorbidity summary scores, such as the OCI, have been suggested as indicators of clinical prognosis because of their predictive ability [7]. Because of this, we reasoned that using the OCI to categorize maternal risk may provide a way to simulate clinical decision making to select a sample of women in need of higher acuity maternal care.

To test our hypothesis, we used a net benefit analysis. Net benefit analysis uses the sensitivity and specificity of a selection method but, unlike an area under the curve analysis, includes the difference in value between the benefit of correct identification and harm of misclassification, known as the exchange rate [8]. The exchange rate allows comparison of the trade-off that occurs as you reduce specificity to increase sensitivity and in this way helps to determine when a model is accurate enough to be useful. Accounting for this trade-off is important for outcomes such as severe maternal morbidity and mortality because the outcomes are so rare, it is possible to have a model with greater than 98% accuracy by identifying all women as low risk. We used an exchange rate that matched the statistical threshold for the model, that is the probability of being identified as high maternal risk by that model. This is appropriate for epidemiologic research because it represents the exchange rate that must be accepted if the model is used [8, 9].

The purpose of this study was to compare the net benefit of assigning a sample of women in administrative data as being at high maternal risk using the OCI method or the conventional method based upon ascertaining the presence or absence (without weighting) of comorbid conditions.

## Methods

This study was conducted as a secondary analysis of administrative data using a net benefit approach.

### Data source

Using data files obtained from the Georgia Department of Public Health for the years 1999 through 2012, we constructed a retrospective cohort by deterministically linking hospital discharge data, supplied to the Georgia Department of Public Health from the delivery hospitals, for all singleton delivery hospitalizations to birth, fetal death, and maternal death certificates using a unique maternal identifier embedded in the files by the Georgia Department of Public Health Office of Health Indicators for Planning. The linking methodology has been previously described [10]. All data was obtained with the permission of the Georgia Department of Public Health and the procedures were approved by the Emory University Institutional Review Board.

### High risk identification models

Two models for identifying women at high maternal risk were used to create two unique samples.

The experimental sample was created using the weighting of 20 comorbid conditions included in the OCI [6]. The index provides a score by summing individual weights for each condition, the weights having been derived from the beta coefficient in the model. The score has been validated to improve the prediction of maternal end organ damage compared to the Charlson Comorbidity Index, and the OCI has been validated with hospital discharge data in a separate sample [6, 11]. Scores for the OCI in these data ranged from 0 to 12 with a mean score of 0.55 (SD 0.90). This was different from the score range from 0 to 19 and mean score of 0.91 (SD 1.42) in the validation cohort when the OCI was created [6].

To use the OCI as a sample selection tool, a cut-off value to indicate high maternal risk was selected by finding the highest net benefit using a score of 2 or higher. As this was the first test of the method, the cut-off value was selected using the population available in these data. While this method prevents generalizability of the cut-off value, it was considered appropriate because the goal of this study was to test the usefulness of the method for sample selection, not validate the cut-off value. The cut-off value to indicate high maternal risk status was selected using net benefit analysis as defined for the main analysis. The cut-off with the highest net benefit was a score of four (Net Benefit of 6 per 100,000); cut-off values less than four had negative net benefits while cut-off values greater than four became progressively closer to zero (See Table 1).

**Table 1:** Results of net-benefit analysis to select a cut-off value for the obstetric comorbidity index

Cut-off value	Sensitivity	Specificity	Positive predictive value	Net benefit per 1000 live births
2	0.52	0.87	0.02	− 185.7
3	0.38	0.96	0.04	−0.17
4	0.16	0.99	0.06	0.63
5	0.16	0.99	0.08	0.33
6	0.03	0.99	0.09	0.12
7	0.01	0.99	0.12	0.05

The comparison group was created using dichotomous identification of any comorbid condition included in the OCI. Dichotomous identification of any comorbidity on a list is the method currently used to stratify fetal and maternal risk in the literature, though the specific list of conditions varies between studies [12, 13]. By applying the conventional practice with the same conditions used for the OCI, this study compared the value of identifying risk with the index summary score rather than comparing the comorbid conditions.

Comorbid conditions were identified using The International Classification of Diseases, Ninth Revision, Clinical Modification (ICD-9-CM) diagnostic codes in the hospital discharge record, which is consistent with prior literature addressing high maternal risk. The authors of the OCI provided the full list of included ICD-9-CM codes in their publication [6]. These codes were used without alteration for the experimental group and the control group.

### Predicted outcome

The predicted outcome for this study was poor maternal outcome which was defined as either severe maternal morbidity or maternal mortality. These data allowed identification of severe maternal morbidity during delivery hospitalization using the hospital discharge record, while maternal mortality was identified using the death certificate and included deaths up to 42 days postpartum.

Maternal mortality is the death of a woman during pregnancy or the postpartum period. For this study, maternal mortality was limited to direct obstetric deaths as defined by the World Health Organization and identified by International Statistical Classification of Diseases and Related Health Problems, 10th revision (ICD-10) codes on the maternal death certificate [14]. The use of direct obstetric death allows a reproducible measure of maternal mortality beyond delivery hospitalization and is limited to deaths related to pregnancy.

Severe maternal morbidity was calculated using a standard algorithm that identifies maternal end organ damage from ICD-9-CM diagnosis and procedure codes [15]. This algorithm updated previous lists of codes that identified specific complications and used length of stay less than the 90th percentile to eliminate diagnosis codes that may have been used to “rule out” conditions. When compared to the gold standard of medical record review, this method had a sensitivity of 77% for identifying severe maternal morbidity [16]. The most common problem with this algorithm is that the ICD-9-CM code for transfusion has a high rate of false positive because it is unable to discriminate between the presence of any transfusion and the presence of a transfusion of four units. This difference is important because transfusion of at least four units indicates severe maternal morbidity in the algorithm. To prevent overestimation of severe maternal morbidity, this study did not include the ICD-9-CM code for transfusion in the severe maternal morbidity algorithm. A sensitivity analysis was performed that included the ICD-9-CM code for transfusion to identify the potential extent of underestimation due to this change in calculation.

### Analysis

The samples created by each method were described by the number of women identified as being at high maternal risk, along with the method’s sensitivity, specificity, positive predictive value, accuracy, and odds ratio for a poor maternal outcome.

The samples were compared for their ability to create a useable research sample of women whose physical condition would warrant transfer to a higher level of maternal care. The comparison was performed with net benefit because, unlike assessment of accuracy or area under the curve, net benefit analysis does not assume the benefits and risks of misclassification are equal [9]. Net benefit was calculated using the formula

$$ Net\  Benefit=\frac{True\ Positives}{n}-\frac{False\ Positives}{n}\left(\frac{pt}{1- pt}\right) $$

where *n* is the total population from which the sample is being selected and *pt* is the probability of being identified as high risk. In net benefit analysis, the method with the highest net benefit is considered “superior.” A model in which no woman is identified as high maternal risk is represented by a net benefit of zero; so any model with a negative net benefit indicates that model performs worse than identifying no woman at high risk [17].

## Results

### Description of data

The study sample for years 2008–2012 included 550,237 unique delivery hospitalizations. The mean maternal age was 27.04 years (SD 6.1) and the mean gestational age at delivery was 38.7 weeks (SD 2.2). Of these hospitalizations, 2654 (0.5%) were identified as having a poor maternal outcome.

The most common conditions identified by the OCI were previous cesarean delivery (17%), age 35–39 years (9.4%), and gestational hypertension (4.5%). The sensitivity of each comorbidity at predicting poor maternal outcome ranged from 0.6% for previous cesarean delivery and gestational hypertension, to 22.6% for chronic congestive heart failure. Full description is available in Table 2.

**Table 2:** Distribution of conditions included in the obstetric comorbidity index among women delivering in Georgia, 2008–2012

	Total *n* = 550,237	Rate per 1000 deliveries	Women with poor maternal outcome *n* = 2654
Previous cesarean delivery	93,634	170.2	648 (24.4%)
Maternal age (years)			
Older than 44	673	1.22	12 (0.4%)
40–44	12,499	22.72	136 (5.1%)
35–39	57,879	105.19	429 (16.2%)
Gestational hypertension	24,632	44.77	150 (5.7%)
Mild or unspecified preeclampsia	16,350	29.71	332 (1.2%)
Asthma	12,159	22.10	87 (3.2%)
Severe Preeclampsia or Eclampsia	7062	12.8	550 (20.7%)
Pre-existing diabetes mellitus	4638	8.43	87 (3.3%)
Drug abuse	3947	7.17	39 (1.5%)
Pre-existing hypertension	2471	4.49	100 (3.8%)
Placenta previa	2439	4.43	117 (4.4%)
Cardiac valvular disease	2073	3.77	32 (1.2%)
Chronic renal disease	1258	2.29	81 (3.1%)
HIV	974	1.77	7 (0.3%)
Sickle cell disease	875	1.59	114 (4.3%)
Systemic lupus erythematosus	601	1.09	14 (0.5%)
Multiple gestation^a^	588	1.07	7 (0.3%)
Congenital heart disease	374	0.68	12 (0.5%)
Alcohol Abuse	272	0.49	4 (0.2%)
Pulmonary hypertension	88	0.16	13 (0.5%)
Chronic ischemic heart disease	71	0.13	2 (0.1%)
Chronic congestive heart failure	31	0.05	7 (0.3%)

^a^Data was limited to singleton delivery by birth certificate

### Model characteristics

The experimental sample, using the OCI, identified 7260 (1.3%) women at high maternal risk. This compared to 193,247 (35.1%) women identified at high maternal risk in the control sample by unweighted OCI. The samples varied greatly in their sensitivity and specificity, though both models had a low sensitivity. The experimental sample had the lowest sensitivity (16.4%) but the highest specificity (98.7%) and was the most accurate (98%). Full model characteristics are available in Table 3.

**Table 3:** Accuracy and net benefit of high maternal risk models to predict severe maternal morbidity or direct obstetric death

	Obstetric comorbidity index	Unweighted comorbidities
Total High Risk	7260 (1.3%)	193,247 (35.1%)
True Positives	436	1588
Sensitivity	16.4%	59.8%
Specificity	98.7%	65%
Accuracy	98%	65%
Positive Predictive Value	6%	0.8%
Negative Predictive Value	99.6%	99.7%
OR (95% CI)	15.6 (14.0, 17.3)	2.8 (2.6, 3.0)
Net Benefit	0.7	−185.7

In the net benefit analysis, the experimental sample created using a cut-off value with the OCI (net benefit 0.7) was superior to the comparison sample built using the conventional sample selection. The control model resulted in negative net benefit which indicated it created a research sample that was worse than identifying no women as high risk. The OCI with a cut-off value of 4 remained the superior risk identification method in the sensitivity analysis.

## Discussion

To our knowledge, this is the first study that compared the value of methods to create a sample of women at high maternal risk in administrative data. In this analysis, the experimental sample created with the OCI using a cut-off value of 4 had a positive net benefit. This appears to be due to the high specificity of the weighted scoring of the OCI. The conventionally derived sample had a negative net benefit, which means it was less valuable than creating a sample by identifying no woman as having high maternal risk. In the case of a research sample, a negative net benefit means it is likely that the harm due to misclassification of women as having high maternal risk outweighed the benefit of correctly identifying women at high maternal risk. Misclassification bias would skew the result of any study toward no difference, and therefore increase the risk of a Type II error.

Though the conventional method of sample selection had better sensitivity, the low specificity of this method resulted in high proportions of women classified at high maternal risk. Given that only 0.5% of women in these data experienced a poor maternal outcome, identification of 35% of the women as being at high maternal risk is likely the result of overestimation of risk status. Though the women identified with these methods may have had an elevated risk, it is unlikely such high proportions of women would be transferred to the highest acuity care to prevent maternal complications. It is more likely most of these women represent a category of moderate maternal risk rather than high risk.

Categorization as women at moderate maternal risk may better simulate clinical reasoning about care than a dichotomous categorization as either low or high risk. Women at moderate maternal risk likely received a higher level of surveillance with their obstetrician rather than being transferred to a sub-specialty practice. In these data, the OCI identified 190,672 (35%) women who might be considered moderate risk; that is, they had a comorbid condition but did not meet the threshold for high maternal risk identification. Stratification of administrative data into low, moderate, and high maternal risk provides an opportunity to better understand the implications of health system level interventions to prevent maternal morbidity and mortality.

In these data, the conventional model identified 35% of the sample as being at high maternal risk but captured 60% of the poor maternal outcomes. This is concerning as it suggests 40% of the severe maternal morbidity and mortality occurred in women with no coded ICD-9-CM risk factors. It is beyond the ability of this study to determine if risk factors were identified by clinicians but not coded in the hospital discharge record. Additional work is needed to improve identification of maternal risk factors in administrative data.

The low positive predictive value of both models indicates poor overall performance which limits the clinical usability for identification of women at high maternal risk in this population. However, the high specificity of the model suggests the potential for use in clinical identification of women at low maternal risk. Interpretation of the predictive value of a model depends on the underlying prevalence of the condition in the population. The low prevalence of maternal morbidity and mortality in this population results in a lower positive predictive value and higher negative predictive value than may be identified in populations with a higher prevalence of poor maternal outcomes.

### Limitations

The methods tested in this paper rely on ICD-9-CM codes, which may not reflect the true distribution of comorbid conditions or clinically important outcomes in the community. Additionally, ICD-9-CM codes are an imprecise representation of the clinical condition of a patient, which will affect both scoring of risk and identification of clinical outcomes. For example, administrative data records only include that gestational diabetes was present, not if it was well controlled. This limitation was considered acceptable for this study because these limitations exist in all administrative data sets, but administrative data is necessary for population level studies. While this method was found to be useful for defining research samples, the results from these data are not generalizable to risk identification in clinical practice and should not be considered a validation for control of confounding or in clinical use.

This study was delimited to risk identification methods that relied on comorbid conditions already known to increase maternal risk, and validated as part of the OCI. Other maternal characteristics are believed to increase risk, but were not included in this study because this study tested a method of sample selection with a validated index. It is possible another risk identification model not tested in this study, such as the Pregnancy Risk Score System, is superior to the OCI as a sample selection tool [5]. It is also possible that inclusion of other criteria, not included in the risk identification model used, would create a superior model.

To use the OCI as a sample selection tool rather than to control for confounding, a cut-off value was selected. The cut-off was selected using net benefit analysis to ensure the comparison of the value of the index was not hindered by assigning a random cut-off. Though the cut-off of 4 was superior for these data, this cut off is unlikely to be generalizable. These data were limited to singleton deliveries and appeared to include underreporting of some outcomes such as alcohol abuse. In these data, although singleton delivery was identified using the birth certificate, 588 records were identified as multiple gestation through ICD-9-CM code in the linked discharge record. These data are not able to determine which source of data, hospital discharge or birth certificate, was accurate in these cases. Underreporting of alcohol abuse has been reported previously, which suggests this variable may be an inherent limitation to use of the index in administrative data [11]. It is likely that the distribution of risk scores, and potentially the superior cut-off value, would be different if these data included all multiple deliveries or had different patterns of comorbidity reporting. Researchers using sample selection with the OCI should assess the best cut-off value for the study data and report the cut-off used and rationale until a standard is determined.

This study relied on net benefit analysis because it allowed comparison of the accuracy of the model while accounting for the unequal value of true positives and false positives when trying to minimize misclassification bias. Accounting for unequal value is the reason the OCI was superior for sample selection despite a lower sensitivity. Net benefit analysis was useful for comparing models for sample selection, but did not assess the diagnostic accuracy of the models. Diagnostic accuracy, as measured by the area under the curve, treats true positives and false positives as equal and therefore would have a different result. The results of this study should not be interpreted as a validation of the OCI for diagnostic reasoning.

Finally, this study compared methods of risk identification by assessing the accuracy of the method at identifying women who had a poor maternal outcome. However, the goal of identifying women at high maternal risk is to ensure they receive the necessary care to prevent poor maternal outcomes. This study is not able to account for the care women did or did not receive that, if controlled, may alter the value of the models. This limitation was considered acceptable because these data represent clinically identified risk and the associated outcomes with the available medical practice.

## Conclusions

The present findings highlight the usefulness of the OCI as a tool to select a sample of women at high maternal risk in administrative data. The OCI was superior to the conventional practice of identifying women with any comorbid condition. Additionally, the OCI allowed identification of a group of women at moderate maternal risk; that is women with identified risk factors but not likely to need transfer to the highest acuity care to prevent poor maternal outcomes. This improved stratification of risk can prevent misclassification bias. Researchers who use the OCI should be aware of the potential for underreported conditions and therefore select the cut-off based on the data being analyzed.

Of particular concern to the authors is that the comorbidities used to identify risk in these models were unable to achieve higher than 60% sensitivity. This is an important finding as it may indicate either the current understanding of maternal risk or the current recording of comorbid conditions in administrative data are not precise enough to create a standard research definition that will prevent misclassification bias during sample selection. In addition to the lack of clinical specificity, the variation in rate of false positives and false negatives for ICD-9-CM codes during delivery admissions provide additional challenges to using administrative data [18]. There are likely non-random reasons for both missing and mis-coded ICD-9-CM codes that may hinder interpretation of findings when risk is measured by ICD-9-CM coding. Further research is needed to identify the best list of comorbidities or other maternal characteristics that predict maternal high risk.The conventional method of maternal risk stratification grew from improved understanding of characteristics associated with poor maternal outcomes. These characteristics are classified as “risk factors” due to increase in relative risk and used to define high maternal risk in administrative data without consideration of the absolute risk. When applying definitions of maternal risk to sample selection in administrative data, understanding how inclusion of additional criteria decreases specificity, accuracy, and net benefit of maternal risk identification is important to preventing misclassification bias. Future research should continue to compare the usefulness of maternal risk stratification methods for administrative data, comparing net benefit of the methods in different study populations and evaluating the impact of misclassification of risk on estimation of benefit for population level interventions.

## Data Availability

The data analyzed in this article are not publically available. They were made available for use through agreements with the Georgia Department of Public Health.

## Abbreviations

ICD-10: International Statistical Classification of Diseases and Related Health Problems, 10th revision
ICD-9-CM: The International Classification of Diseases, Ninth Revision, Clinical Modification
OCI: Obstetric comorbidity index
